# Supplementary material for: Are carbon emissions trading and green financial instruments synergistic? -Comprehensive quantitative research based on content analysis
Source: PLoS One. 2024 Mar 7;19(3):e0298601. doi: 10.1371/journal.pone.0298601 (PMC10919601; doi:10.1371/journal.pone.0298601)
Supplement: S1 Data — (ZIP) [file pone.0298601.s001.zip › Data/地级市Z_F工作报告.zip!/░▓╗╒/╗┤─╧╩╨/└┤╫╘╣1⁄2╓┌║┼í╛┬φ┐╦╩2╛▌═°í┐_2009.docx]

各位代表：

  现在，我代表市人民政府，向大会报告工作，请予审议，并请市政协委员和其他列席人员提出意见。

**一、2008年工作回顾**

  刚刚过去的2008年，是我市发展改革进程中很不平凡的一年。全市人民在市委的坚强领导下，全面贯彻党的十七大、十七届三中全会和胡锦涛总书记视察安徽时的重要讲话精神，深入学习实践科学发展观，积极克服自然灾害带来的影响，切实应对国内外形势发生的变化，攻坚克难，扎实工作，保持了经济社会又好又快发展的良好势头，圆满完成了市十四届人大一次会议确定的年度目标任务。

  ——经济跨上新台阶。预计，全市生产总值449亿元，增长15.7%；财政收入60亿元，增长23.7%；固定资产投资225亿元，增长10.4%；社会消费品零售总额134亿元，增长22.8%；外贸进出口总额1.3亿美元，增长41%。

  ——城乡面貌发生新变化。城市化水平达到46%以上，新增城市道路面积67.5万平方米、绿化面积146万平方米。万元生产总值能耗下降4.2%，二氧化硫、化学需氧量削减超额完成省下达的目标任务。新农村建设扎实推进，城乡环保一体化全面启动。山南山北联动发展取得突破，合淮阜高速公路建成通车，合淮同城效应明显增强。

  ——社会事业取得新进步。高中阶段毛入学率69.4%；新型农村合作医疗平均参合率86%；城镇居民医保超额完成省下达52万人的目标任务；城镇和农村居民最低生活保障面分别为6.9%、3.9%；人口自然增长率6.9‰。

  ——人民生活得到新改善。城镇登记失业率4.4%，新增城镇就业3.2万人。预计城镇居民人均可支配收入1.3万元，增长8.3%；农民人均纯收入4500元，增长20%；居民消费价格指数105.3，低于全省全国平均水平。

**一年来，我们重点抓了以下工作：**

  （一）优化产业布局，加快工业强市步伐。全年新增规模以上工业企业54户。预计规模以上工业实现增加值240亿元，增长27%；实现利税69.8亿元，增长60.3%。启动了“T”型工业布局研究和导向规划，开展了合淮工业走廊规划编制，倾力打造沿淮工业带，推进市开发区、煤化工新区、凤台开发区、淮南工业园和12个工业集聚区的扩容和规划。煤电基地综合生产能力大幅提升。预计原煤产量6700万吨，增长43.4%；发电量460亿千瓦时，增长64.1%。望峰岗煤矿、洛河电厂三期、凤台电厂一期、潘三煤矸石电厂等一批重大项目建成投产，500千伏西通道工程淮南段顺利竣工。煤化工基地加快推进，煤化工新区破土动工。淮南煤气化技术攻关取得重大突破，煤基合成油催化剂、甲醇制丙烯工业试验装置等拥有自主知识产权的科技先导型项目建成试生产，100万吨纯碱二期项目开工建设。加工制造业加快发展。景丰高档卷烟纸、益益乳业万吨配方奶粉、电子工业园、海底光缆及连接器等项目竣工投产，医药物流园、中润集中供热等项目开工建设。实施千万元以上工业技改项目30个。

  高度重视节能减排工作。我市被列为全省6个推广高效节能照明试点市之一。建成16个污染减排重点项目，东部生活垃圾处理场主体工程完工，西部污水处理厂、凤台污水处理厂、龙王沟泵站扩容工程等建成投入运行。环保专项行动获得国家表彰，田家庵区荣获全省环境保护模范城区。

  （二）加强城市建设管理，推进合淮同城化。坚持规划先行，完善山南新区各项规划，完成西部城区和潘集区分区规划，完成21个控制性详细规划和7个专项规划编制，基本完成滨河城市设计、高塘湖滨水地区规划、八公山风景区规划、舜耕山风景区概念性规划，着力彰显山水林城融为一体的城市特色。

  加大城市建设力度，巩固和发展山南、泉大、城投等建设模式。荣获2008年度中国城市科学发展创新奖。山南新区建设步入快车道。洞山隧道、淮河大道建成通车，高压走廊改造、农民新村一期等工程竣工，市级机关办公楼、武警支队机关新营区开工建设。淮河四座大桥、淮南新港等项目前期工作加快推进，淮蚌高速公路、206国道改线、东西部第二通道、夏郢西路等开工，龙湖南路一期、淮河大桥维护及南引线工程建成通车。旧城改造继续推进。全市新开工房地产开发面积190万平方米，竣工125万平方米。龙湖路商圈加速提升，合肥百大淮南商厦、华联二期、新百二期、商之都淮南店等建成开业。创建中国优秀旅游城市通过省级初验，新增国家3A级旅游景区2个。启动龙湖公园改造，新建游园4处，完成植树造林2万亩。文明创建活动深入开展，数字城管积极推进，城市综合管理水平进一步提高。经过充分酝酿、认真准备，多方协调配合，制定和实施了一系列政策措施，依法取缔正三轮机动车非法客运、切实保障残疾人基本生活工作取得明显成效。

  全面推进合淮同城化。基本完成合淮同城化总体规划编制，积极开展区域生态、城际交通、信息通讯、现代服务等专项规划编制，共同签定了商贸、旅游、信息、交通等10项合作协议，共同举办了区域发展暨合淮同城化高峰论坛，两市交流日益频繁，两市合作不断扩大。

  （三）坚持统筹发展，促进城乡一体化。全面落实强农惠农政策，制定了加快农业和农村发展实施意见、农业产业化“712”提升行动计划。全市财政用于“三农”支出11.6亿元，比上年增加2.5亿元。

  粮食总产达132万吨，增长9.4%，创历史新高。新增省级农业产业化龙头企业1家、国家级农业标准化示范区2个、绿色食品8个、有机食品1个、无公害农产品13个。深入推进“万村千乡市场工程”，建成农家店95个，配送中心1个。完成12个农贸市场的升级改造任务。村镇布点规划和夏集、上窑、曹庵小城镇规划完成，新农村建设“1860”示范工程涌现出一批亮点。“一绿二清五改”村庄整治活动深入开展，建成养沼一体化大型沼气池6个、小型沼气池2160个。治淮工程取得突破性进展，洛河洼、石姚段行洪堤退建及加固工程开工，董峰湖、汤渔湖行洪堤加固等工程竣工。

  加快发展城乡社会事业。全市财政用于教育、卫生和文化体育事业的支出分别为88986万元、29226万元和5263万元，比上年增长19.5%、26.8%、23.9%。推动教育均衡发展，完成城市中小学布点规划，省级示范高中、市级特色示范学校创建和农村中小学达标评估工作取得新成效。职业教育和高等教育加快发展。医疗卫生服务体系不断完善。全国第二批城镇居民基本医疗保险试点市工作扎实推进。实现失业保险市级统筹。市食品药品检验中心主体工程、市第一人民医院改扩建主体工程完工。妥善处置三鹿奶粉事件，及时免费筛查救治患儿。荣获全国“无偿献血”先进城市称号。推进文化大市建设。一县一乡被命名为“全国民间文化艺术之乡”。全国第三次文物普查全面展开，列入国家级非物质文化遗产名录2项。《八公山志》再版发行。博物馆二期工程、家乐福横店影视城加快建设，建成农家书屋140个。积极推进数字电视试点网络改造，有线电视播控中心大楼建成。市全民健身中心开工建设，成功承办省第二届体育大会。顺利完成569个村委会换届选举，积极开展第二届社居委换届工作。人口计生、统计、档案、地方志、防震减灾取得新进步，妇女儿童、老年人、残疾人权益得到保障，民族、宗教工作取得新进展，外事、对台和国家安全工作进一步加强，国防动员、人民防空、民兵预备役建设迈上新台阶。

  （四）实施“三位一体”突破，全面推进“9771”提升行动计划。立足打基础、利长远、增后劲，千方百计抓好项目、投资、招商工作。全年新开工项目450个、竣工项目280个，分别增长5.9%、11.6%。“9771”提升行动计划项目库储备规模达2000多亿元。建立重点项目市级领导联系人制度，加强项目投资月度分析。调整了市开发区、煤化工新区、山南新区优惠政策，出台了优化投资环境办法，组建了外商投资服务局。成立招商小分队67个，开展了小分队招商、专业招商和节会招商。成功举办第十五届中国豆腐文化节，组织参加了徽商大会、厦洽会等大型系列节会，共签约项目253个。全年引进内资115亿元，实际利用外资1.1亿美元。对外合作水平进一步提升，与省电信公司签订了“数字淮南”信息化建设战略合作协议，与环保部南京环科所签订了全面加强科技合作框架协议，与省农垦集团签订了合作开发淮南农场协议。

  制定和实施一系列政策措施，加快非公经济和县区域经济发展。全市个体工商户总数达34693户，新增4745户；实现增加值11265万元，增长8%。凤台县财政收入继续位居全省县级首位，财政收入增幅超过全市平均水平的县区达到5个。

  积极应对金融危机，认真贯彻国家和省扩大内需、增加投资的各项政策，出台了促进经济平稳较快发展保障改善民生20条、加快非公和县区域经济发展50条、促进房地产业发展11条、推进自主创新24条等一系列政策措施。安排8300万元贷款贴息资金、3000万元科技专项资金、2000万元中小企业贷款风险补偿基金、500万元项目编制补助和激励资金。取消和停征73项行政事业性收费，降低15项经营性服务收费标准。全年增值税转型试点退税10.6亿元，争取国家年内新增投资2.9亿元，为当前及今后一段时期发展打下了良好基础。

  （五）推进改革创新，破解发展难题。完成8户国有企业改革和集体林权主体改革，成立了市金融服务中心，市邮政储蓄银行开业。广泛建立市厅会商机制，取得积极成效。土地报批取得新成绩。全年上报单独选址和批次用地3万亩，上报土地置换3000亩，完成新增耕地占补平衡1.1万亩。积极破解融资难题，理顺了城市建设投资公司体制机制，获国家开行授信71亿元。组建了产业投资公司。成立市担保投资有限公司。广泛开展银企对接活动。自主创新能力进一步增强。再次荣获全国科技进步先进市，毛集实验区荣获国家可持续发展先进示范区，淮南矿业集团被命名为省级自主创新型企业。全年鉴定科技成果40项，重新认定高新技术企业11家，列入国家各类科技计划项目7个，获得国家创新基金项目1个。成功举办第二届皖北区域（淮南）产学研对接会，对接项目217个。质量兴市扎实推进。新创安徽名牌产品6个，八公山豆腐获得国家地理标志产品保护。深入实施人才强市战略，人才队伍不断发展壮大。

  （六）保障和改善民生，维护社会稳定。全面实施18项民生工程，共投入财政资金4.7亿元。农村低保实现扩面提标，农村五保供养率达100%。义务教育经费保障机制、高校和中职学校困难家庭学生资助体系惠及30万名学生，全部消除2005年入库的144个D级危房项目。新型农村合作医疗实现全覆盖，城乡医疗救助制度得到调整，重大传染病救治救助扎实推进，改扩建乡镇卫生院9个、村卫生室62个、社区卫生机构32所。城镇未参保集体企业退休人员基本生活保障按时足额发放。完成11万人农村安全饮水工程建设任务。实施白内障患者复明手术660例。累计奖励农村计划生育家庭扶助对象884人。发放大中型水库移民补助资金44.5万元。建成“村村通”工程99.4公里、农村客运站9个。建成经济适用房13万平方米、廉租房2500平方米，发放廉租住房补贴1017.6万元。大力推进采煤沉陷区居民住房建设，开工建设安置新区58.1万平方米，竣工28.2万平方米，安置居民4550户，建成过渡活动板房1200套。深入开展“隐患治理年”活动和“安全生产百日督查专项行动”，安全生产形势总体平稳。加强信访工作，深入开展矛盾纠纷排查、重信重访案件专项整治等活动，北京奥运会、残奥会期间信访稳控工作扎实有效。加强物价监管，整顿和规范市场经济秩序取得成效。重拳整治非法传销，捣毁窝点320处，解救人员860人。平安淮南建设取得新进展，一县两区被命名为省级平安县区。扎实推进公安“三基”工程建设，社会治安综合治理基层基础不断夯实。依法打击各类犯罪活动，有效维护了公共安全和社会稳定。

  （七）深化管理服务，强化政府自身建设。认真开展深入学习实践科学发展观和解放思想大讨论活动，围绕制约淮南发展的突出问题，开展专题调研。修订完善《政府工作规则》，规范政府行政行为。认真开展“两集中、两到位”试点，切实完善市县乡三级联动服务体系。提请市人大常委会审议地方性法规议案4件，制定政府规章4件。在全省率先建立了乡镇政府法制员工作制度，调处矛盾纠纷2338起，办理行政复议案件38件、各类法律援助989件。取消行政审批项目19项。保留规范性文件106件。自觉接受市人大及其常委会的法律监督和市政协的民主监督，办理人大代表议案2件、建议125件，办理政协委员提案489件。扎实开展反腐倡廉制度建设推进年活动，切实落实党风廉政建设责任制。加强行政监察和审计监督，完善重大决策咨询、听证、公示等制度，努力打造阳光政府。

  在过去的一年里，我们夺取了抗雪救灾和灾后重建的全面胜利，筹措资金731.8万元，全面完成849户、1903间雪灾倒房建设任务。我们积极支援抗震救灾，捐款捐物合计4800余万元，运送救灾帐篷700顶，派遣救援人员46名，为灾区送去了淮南人民的深情厚谊。我们积极有效应对春夏之交发生的手足口病严峻疫情，立足早防、早控、早治，全面落实防控措施，全市无一例死亡病例发生，被省政府授予防控手足口病先进集体称号。我们成功组织了奥运圣火传递活动，240万淮南人民共享圣火荣耀，“五彩淮南”绽放传递激情，充分展示了淮南经济发展、社会和谐、民族团结的良好形象。

  这些成绩的取得，是省委、省政府和市委正确领导的结果，是市人大和人大代表、市政协和政协委员关心支持的结果，是全市人民共同拼搏的结果。在此，我代表市人民政府，向全市人民及驻淮部队、武警官兵、公安民警，向各民主党派、工商联、无党派和各界人士，向关心支持淮南现代化建设的驻淮单位及海内外朋友，表示衷心地感谢！

  在看到成绩的同时，我们也清醒地看到经济社会发展中的矛盾和问题，清醒地看到我市当前发展面临的挑战和风险。突出表现为：一是经济结构性矛盾仍很突出，非公经济和县区域经济发展不足，经济外向度偏低；二是自主创新能力不强，节能减排和环境保护压力不断加大；三是受金融危机的冲击和影响，部分行业产值和效益下滑，部分企业生产经营遇到困难，对经济增长、财政收入、就业稳定等方面产生的影响逐步加深。四是涉及群众切身利益的问题有待进一步解决，农村基础设施建设相对落后，社会事业发展不足，规划管理滞后；采煤沉陷区治理矛盾突出，棚户区改造任务难巨；低收入家庭生活困难，社会保障水平有待提高；安全生产形势不容乐观；信访稳定的压力加大。五是发展环境不优的问题比较突出，强买强卖、乱搭乱建等现象较为严重；一些部门大局意识不强、办事效率不高、工作落实不够，官僚主义、形式主义和奢侈浪费现象还不同程度存在。对此，我们务必高度重视，认真加以解决。

**二、2009年工作总体要求**

  2009年，是推进科学发展、加速淮南崛起、实现“十一五”目标任务的关键一年。积极有效应对金融危机带来的影响，扎实做好今年的各项工作，千方百计保持来之不易的发展势头，是我们面临的最大挑战，也是最核心的任务。当前这场百年不遇的全球金融危机还在演变，我国经济增速下滑的压力更加突出，我们必须做好应对更加困难局面、迎接更加激烈竞争的准备。同时，我们也应当看到，我国发展的重要战略机遇期没有改变，我市经济平稳较快发展的基本面没有改变，淮南厚积薄发、加速崛起的阶段性特征没有改变。国家已经并将继续出台一系列强有力的措施，给我们带来了很多机遇。我们必须坚定信心，知难而进，化危为机，加快发展。今年政府工作的基本思路是：以邓小平理论和“三个代表”重要思想为指导，全面贯彻党的十七大、十七届三中全会、中央、省经济工作会议和市委八届八次全会精神，深入落实科学发展观，围绕“推进科学发展，加快淮南崛起”这一主题，大力实施“七大战略”和“9771”提升行动计划，立足扩大内需，强化投资拉动，推进结构调整，转变发展方式，深化改革开放，保障改善民生，努力实现经济社会平稳较快发展。

  今年经济社会发展的主要预期目标是：地区生产总值增长11%以上，财政收入增长12%，固定资产投资增长20%以上，社会消费品零售总额增长12%，城镇居民人均可支配收入增长7%，农民人均纯收入增长7%，新增城镇就业岗位3万个，城镇登记失业率控制在5%以下，人口自然增长率控制在7.5‰以内，单位生产总值能耗下降4.38%，二氧化硫排放量下降1%，化学需氧量排放量下降2%。

  面对新形势、新任务，我们必须坚持把扩内需、保增长作为首要任务。今年统筹投入各类财政性资金60亿元，安排重点项目投资160亿元，带动社会投资，力争实现全社会固定资产投资270亿元以上。我们必须坚持工业化和城市化双轮驱动，对外，加快合淮同城化，参与泛长三角区域发展分工与合作，加速国际化进程；对内，加快城乡一体化，做精一产、做强二产、做大三产，做优做美城市。我们必须坚持把提高自主创新能力、加快发展方式转变摆在更加突出的位置，既要在激活存量、扩大增量上做加法，又要在节能减排、环境污染上做减法；既要关注税收经济、就业经济，又要关注生态经济，切实提高发展质量和效益。我们必须坚持把实现好、发展好、维护好人民群众的根本利益作为一切工作的出发点和落脚点，无论形势怎么变，根本宗旨不能变，切实把老百姓的事情办好，真正让人民群众满意。

**三、千方百计扩内需保增长**

  积极贯彻和落实进一步扩大内需促进经济增长的政策措施，把加大投资与促进消费结合起来，把促进即期增长与增强发展后劲结合起来，切实提高经济抗震减压、抵御风险能力。

  千方百计保持投资较快增长。统筹安排工业投资100亿元以上，强化工业投资对经济增长的带动力和支撑力。理顺城市分级建设、管理、投入体制，力争全年完成城市基础设施建设和房地产投资100亿元以上。着力实施118个投资亿元以上的重大项目、41个重点技改项目。推进大交通建设，加快淮蚌高速公路、206国道改线建设，推动淮滁高速公路、102省道和308省道改扩建工程前期工作。充分发挥“淮河”优势，统筹推进“五水”建设。推进淮河四座大桥、淮南新港前期工作，力争实施董峰湖行洪区调整改造工程，加快洛河洼、石姚段行洪堤退建及加固工程，推动焦岗湖、高塘湖、西淝河、架河、港河等洼地治理。加强项目编制工作，提高项目质量，对上争取国家政策、资金支持，对外招商引资，对金融部门争取贷款，对土地部门争取土地指标。进一步完善项目推进机制，健全法人负责制和领导联系制度，强化“9771”提升行动计划项目库储备功能，建立工业、城市、农业等固定资产投资项目库，确保今年新增项目储备规模500亿元以上。

  千方百计加大融资力度。发挥财政资金在融资工作中的引导作用，发挥城投、产投、担保公司、小额贷款公司等各类融资平台作用，开展多种形式的银企合作、银政合作，加大向世行、亚行和国外政府贷款力度。加快金融改革，着力构建现代金融体系。启动新型农村金融机构试点三年规划，完善农村金融服务。加快淮南市区联社改革，组建通商农村合作银行。积极引进更多股份制商业银行来淮设立分支机构，加快组建小额贷款公司，力争每个县区1-2家，加快城投公司发债步伐，力争更多有条件的企业发行债券。整合担保资源，做大市担保公司规模，积极争取更多担保公司来淮发展，壮大担保业，规范担保行为。

  千方百计招商引资。突出抓好四件事：一是定任务。今年全市利用外资1.2亿美元以上，增长10%以上，引进内资150亿元以上，增长30%以上。年初将目标任务分解到各县区、各部门。二是搭平台。抓好外商投资服务局建设，安排招商引资专项资金500万元，招商小分队200个以上，重视大企业招商、大项目招商、园区招商、以商招商、产业链招商，加快“走出去”步伐。三是建机制。制定出台招商引资考核奖惩办法，浓厚干群总运动、上下齐招商的氛围。支持在淮企业发展总部经济，重点吸引国内外大企业、大集团来淮设立区域性总部，促进总部经济和驻地经济加快发展。四是优环境。要优化办事流程，减化办事程序。精减审批，减少收费。建立健全首席代表制、全程代理制、限时办结制。

  千方百计刺激消费。完善消费促进政策，增强消费能力，改善消费预期，大力培育商贸、住房、文化、旅游等消费，积极发展与消费升级相关的产业。加快商贸物流专业化、特色化、高端化发展，打造皖西北商贸物流中心。加快家乐福商务广场、红旗商贸文化广场、沃尔玛购物广场、东方国际购物中心、医药物流园建设，着力提升淮矿物流大市场、永安大市场等规模，积极引进乐购、麦德隆等知名连锁企业。健全农村流通网络，扩大农村消费。加大“家电下乡”推广力度，推进“万村千乡市场工程”。完善城市社区便民服务设施，继续推进农贸市场升级改造，满足居民便利消费、放心消费需要。结合旧城改造、棚户区改造、新区开发和行蓄洪区、采煤沉陷区、新矿区移民迁建，促进房地产业健康快速发展。全年施工房地产开发面积400万平方米，竣工120万平方米。落实保障性住房配建制度，建设廉租房2.75万平方米。大力发展文化、旅游业。重点抓好八公山旅游综合服务区开工，推进新四军纪念林陈列馆、焦岗湖渔家乐风情岛、高塘湖中心岛休闲渔业等景区景点建设；积极争创焦岗湖、上窑国家4A级旅游风景区。加快旅游商品开发和服务体系建设，实行多元化营销，实现旅游总收入增长20%。

  千方百计保企业。保企业，就是保增长、保就业、保稳定，必须放在重中之重抓紧抓好。主要采取以下措施：一是实行银企抱团取暖，切实解决企业融资难问题。二是用足用活中小企业贷款风险补偿基金、中小企业贷款贴息资金和开发区、工业园区贷款贴息资金，进一步出台支持扶持工业企业特别是中小企业的若干政策，加强对中小企业的财税支持力度。三是支持企业实行产业转型和结构优化升级，在危机中抢抓商机，在逆境中开拓市场。四是加强和改善对企业的服务。通过企业自救、政府拯救、社会施救，保住发展成果，保证经济持续健康发展。

**四、加快工业化和城市化步伐**

  （一）推进工业强市，着力建设能源重化工业基地

  以加强合淮工业走廊、沿淮工业带“T”型产业布局规划为主线，以加快园区平台建设为重点，以多个工业集聚区为补充，以煤电化、大企业、大项目为牵引，促进扬煤与凸企互动，扬长与补短结合。

  充分发挥资源优势，实现资源就地转化。进一步增强煤电集群效应。开工建设朱集东矿、朱集西矿、杨村矿、平电三期、田集电厂二期、凤台电厂二期、潘集电厂、顾桥煤矸石电厂、1000千伏特高压工程，加快顾桥矿南风井、潘一矿东区安全技改、新庄孜煤矸石电厂等项目建设，确保张集西风井建成投产。加快煤化工基地建设，重点延伸甲醇制烯烃、硝酸、化肥、煤焦化、替代燃料、电石乙炔六大产业链。开工建设60万吨甲醇及20万吨二甲醚项目，加快100万吨纯碱二期项目建设，建成淮化清洁工艺生产项目。积极发展高效载能工业。推进煤-电-铝一体化、煤-电-硅-光伏一体化发展。积极推广煤矸石和粉煤灰综合利用、瓦斯和生物质发电、中水回用等先进技术，抓好宏泰钢铁再生金属循环经济产业园、年产400万吨海螺水泥、凯迪生物质发电等项目建设。积极筹新中国成立家大型煤炭储备库和中国淮南煤炭现货期货交易中心，积极争取直供电试点，推动资源优势向发展优势转化。

  充分发挥园区集聚优势，做好“无中生有”文章。完成市开发区、煤化工新区、凤台开发区、淮南工业园以及12个工业集聚区的扩容规划，促进布局集中、产业集聚、土地集约。积极推进中心城区工业企业退城进园，推进园中园、“飞地”园区等建设，加快发展机械电子、生物医药、新型材料等制造业。加强与省农垦集团合作，推进工业、农产品加工、商住综合开发，打造垦区新农村城镇化试验区，推动开发区新一轮大建设、大发展。开工建设长壁煤机、重庆啤酒、华宫胶管等12个项目，加快景丰纸业二期、汇通纺织二期等11个项目建设，建成电子工业园、康寿医疗器械等10个项目，引进项目20个以上。完善基础设施，创新管理服务，努力把园区打造成为产业集聚的沃土、企业投资的热土、环境优化的净土。

  充分发挥大企业优势，加快企业梯队培植。支持大企业领跑工业化、参与城市化、反哺农业现代化，锻造淮南崛起的脊梁。把培育新企业、加快发展中小企业摆在更加突出的战略位置，促进各类企业茁壮成长，形成“梯队型”结构，全年新增规模以上企业60户以上。

  （二）推进城市大建设，着力打造淮河明珠

  以战略眼光规划城市，以精品意识建设城市，以精细化理念管理城市，以市场化手段经营城市，加快建设山水园林城市、滨河滨湖城市、宜居宜游宜创业城市。

  加强城市规划。统筹山南山北、统筹东部西部、统筹河南河北，着力构建“三山为肺、三水为脉、三城为心”的城市发展格局。完成主城区文化设施、城区路网、综合防灾等专项规划，启动并完成上窑山风景区总体规划、主城区30平方公里控制性详细规划、西部沉陷区生态治理规划，制作主城区三维沙盘，完成淮河大道两侧、山南新区起步区水系景观设计。

  加快山南新区开发。开工建设南纬二路、南纬七路、南经六路等10条道路，全面完成南纬三路二期、北环路等6条道路建设。加快市级机关办公楼、武警支队机关新营区建设，开工建设奥林匹克体育场、农民新村二期、自来水厂、报业大厦、国投新集总部、移动公司生产大楼、职教园区、淮南二中、安徽理工大学新区等20个项目建设，启动科技馆、人民会堂、政务服务中心等规划建设，实施起步区水系景观改造工程。

  完善城市基础设施。加快东西部第二通道建设，推进洞山西路、龙湖路、国庆东路、谢李路等改造，完成洞山中路、陈洞路、金家岭路、夏郢西路、卧龙山路、蔡新路、沿矿路、湖滨东路扩建、广场北路贯通等工程，规划建设朝阳路等3座过街人行天桥。实施城市亮化和景观照明工程，消除主次干道路灯不亮和无路灯问题，开展城市支路、老街巷亮化试点，提高城市亮化水平。实施城市水系整治工程。启动舜耕山风景区核心区、淮河公园、淮西湖公园建设，完成龙湖公园改造，续建十涧湖国家城市湿地公园，推进泉大资源枯竭矿区环境修复。加强城乡绿化，新增城市绿地面积100万平方米。完成植树造林2万亩，建设合淮阜高速公路淮南段森林生态长廊。建立市级生态公益林补偿机制。

  提升城市文化品位。高标准编制文化产业发展规划，促进文化大市建设。深度挖掘淮河文化、煤文化、汉文化，做活“五古一珍”、花鼓灯艺术、少儿艺术等文化品牌，增强文化的表现力、吸引力和感染力。加快公共文化基础设施建设，逐步建立覆盖城乡的公共文化服务体系。高度重视网络文化建设。加快推进山南新区文化园区和万人坑教育馆规划建设，统筹规划淮南大剧院、淮南画院，开工建设西部书城、电影城等项目，建成乡镇综合文化站16个、农家书屋120个。加快文化体制改革，鼓励社会资本投资文化产业，引导文化团体走向市场。加大文化惠民工作力度，认真组织“四进一下”等公益性文艺演出活动。继续做好第三次全国文物普查和非物质文化遗产保护工作。办好第十六届中国豆腐文化节和第二届中国淮南国际少儿文化艺术节。积极备战安徽省第十二届运动会，办好市第六届运动会。开工建设市水上训练基地，建成全民健身综合训练馆。

  加快城市精细化管理。巩固国家园林城市创建成果，加快安徽省文明城市、国家环保模范城市、国家生态园林城市创建步伐，进一步完善“两级政府、三级管理、四级网络”的城市管理体制，健全长效管理机制。建立数字城管系统，推行网格化管理。启动城区杆线整治工程。加大违法建设拆除力度。实施城乡清洁工程，改善城乡环境。巩固依法取缔正三轮机动车非法客运成果，确保不回潮、不反弹。

  （三）推进非公经济更快发展，着力实施县区域突破

  抓住时机利用外资、引进内资、启动民资、扩大融资，壮大非公经济和县区域经济，加快形成拉动内需的支撑力量。

  推动非公经济上台阶。全面贯彻落实推动非公经济和县区域经济又好又快发展的若干意见，研究制定相关配套政策。积极搭建中小企业融资平台，建立健全中小企业信用担保体系，设立1000万元非公经济发展专项资金，重点培育和扶持“小而精”、“小而特”、“小而专”、小而优”的龙头型中小企业，使非公经济在促进经济增长、扩大就业、增加税收和活跃市场等方面，发挥越来越大的作用。力争非公经济的比重达到40%，对财政贡献率提高10个百分点。

  推动县区域经济上水平。坚持分类指导，简政放权，在产业布局、项目安排、基础设施、财政转移支付、税收分成方面向县区倾斜，配套出台分类指导的区域政策，增强县区自我发展能力。发挥凤台、潘集的煤炭资源优势，建好“煤电化”主战场和粮食主产区。发挥田家庵中心区的优势，大力发展现代物流、现代服务等产业，提高首位度。支持大通用好发源地，办好开发区，建好东大门。大力促进西部地区经济发展，支持谢家集、八公山加强西部城区、旅游景区、生态矿区、新型工业集聚区建设。支持毛集国家可持续发展先进示范区建设，推进景区、城区、园区互动发展。

  （四）推进自主创新和节能减排，着力增强发展可持续性

  充分利用市场约束的倒逼机制和全球经济调整的洗牌效应，在提高经济素质上加强自主创新，在节能减排上加大攻坚力度，力求取得更大成效。

  坚持把推进自主创新作为转变发展方式的中心环节。加快创新型淮南建设，努力实现再次进入全国科技进步先进市的目标。认真落实关于推进自主创新的若干意见，加大科技投入，建立稳定增长机制。要让创新优势不断凸显。大力促进传统产业高新技术化和高新技术产业化，突出抓好“四点一园”（即煤炭高效率开采及瓦斯综合利用先进生产力建设，高标准、高产出、大循环火力集中发电，煤化工精深加工及循环利用，生物医药等新兴产业，山南高新技术园区）建设，促进一批拥有自主知识产权技术就地转化。要让创新型企业不断涌现。切实抓好高新技术企业的培育，加强申报重新认证工作，确保新增省级创新型试点企业3家。要让产学研结合不断紧密。积极申报国家火炬计划3项、创新基金项目5项，申请专利315件。开工建设国家煤及煤化工产品监督检验中心。要让创新基地的集聚能力不断增强。加快省级生物医药及高新技术产业基地建设，着力打造矿用电子高新技术产业群，积极创建省级防爆电气优质产品基地。要让创新创业的氛围不断浓厚。强化领军型人才集聚培养，完善风险投资机制，鼓励更多的科技人才自主创业。

  坚持把节能减排与工业化、城市化同部署同推进。突出重点行业、重点企业和重点领域，加快10大重点节能工程实施进度，切实抓好11个污染减排重点项目建设，加快山南新区污水处理厂建设，推进村镇生活污水生态处理工程试点。突出规划、保护、治理、经营等关键环节，加快推进城乡环保一体化。加强农村工业、生活污染和农业面源污染防治，加快农村污水集中处置、垃圾集中收储、农药化肥减量喷施、秸秆综合利用，努力建成一批省级环境优美乡镇和生态村。我们要持之以恒地抓下去，让淮南的山更绿、水更清、天更蓝、城更美。

**五、加速城乡经济社会一体化进程**

  坚持工业反哺农业、城市支持农村和多予少取放活方针，以城乡一体化规划为龙头，着力在产业布局、基础设施建设、公共服务、社会管理一体化等方面取得突破，促进城市基础设施向农村延伸、城市公共服务向农村覆盖、城市现代文明向农村辐射，推动城乡经济社会发展融合。

  （一）加快农村发展，推动城乡经济共同繁荣

  积极发展现代农业。按照高产、优质、高效、生态、安全的要求，加快农业结构调整。强化粮食主产区功能，重点抓好“百万亩”优质专用小麦和“百万亩”优质水稻核心示范区建设，努力打造沿淮“粮仓”。积极发展特色种植业、林木花卉业。大力发展养殖业。抓好养殖水域滩涂规划建设。制定加快奶业发展实施意见，新增奶牛1000头。加快农业规模化、标准化生产，建成国家级农业标准化示范区１个、省级２个，建成生猪、家禽标准化养殖小区各５个。加快实施农业产业化“712”提升行动计划，力争全年农产品加工业产值超过90亿元。争创国家级龙头企业1家、国家级名牌农产品1个，新增绿色食品、有机食品和省级名牌农产品10个以上，龙头企业产值和利税分别比去年增长10％以上。

  切实拓宽农民增收渠道。坚持政策惠农、科技兴农、创业富农，建立农民持续增收的长效机制。健全农业补贴制度，进一步扩大范围、提高标准，逐步加大农民种粮补贴，完善农机农资综合补贴动态调整机制，稳步提高粮食收购价格。健全政策性农业保险制度，提高农业抗风险能力。加大农业科技创新，通过农业结构调整、农业内部挖潜、农业技术推广、农业科技成果转化等措施，提高农产品经济效益。着力完善农村综合服务体系，全年培育新型农村经济合作组织20个，建成高标准农技推广服务站10个，形成产供销一条龙、贸工农一体化的发展方式和增收模式。

  扎实推进新农村建设。全年完成“三农”支出13亿元以上，增长13％。一是推进小城镇改造。重点抓好上窑镇亲山近水型、平圩镇综合服务型、夏集镇农副产品加工创业型规划建设。二是推进老村庄整治和扶贫开发。抓好一批城中村改造、中心村建设、自然村缩并、空壳村搬迁，支持有条件的村庄逐步发展成农村新社区。完成10个贫困村整村推进任务。三是推进村村通工程。建成农村公路200公里、农村客运站10个、候车亭178个。四是推进沼气工程。建成沼气池2万立方米。五是推进农村安全饮水工程。再解决农村11万人安全饮水问题。六是实施通讯工程。积极开展新农村信息化试点建设。七是高度重视采煤沉陷区治理。积极争取河南四区资源型城市转型试点。制定并实施采煤沉陷区综合治理实施方案，突出抓好新村搬迁选址、拆迁补偿和过渡活动板房建设，全面完成2004年开始实施的国家采煤沉陷区综合治理项目建设任务。切实解决好采煤沉陷区居民住房、培训、就业和社会保障问题,是一项极为艰巨的任务。我们要以百倍的努力，把这项关系民生的大事做好。

  高度关注农民工。加强农村职业教育和农民技能培训，大力提高农民素质。加大用工信息发布力度，密切关注动向，及时分类指导，切实提高农民工输出组织化程度。积极引导和扶持有资本、有技术、有经验的农民回乡创办领办企业，化就业压力为创业动力，变返乡潮为创业潮。

  （二）大力发展社会事业，促进城乡基本公共服务均等化

  坚持教育优先发展、均衡发展、规范发展。优先加强教育规划，优先安排教育支出，优先解决农村地区、困难家庭、留守儿童平等就学问题，确保市本级教育支出预算安排增长１３％。大力发展学前教育、特殊教育、义务教育、高中教育、职业教育和高等教育，加快实施城市中小学布点规划，积极整合高中教育资源，合理利用闲置校舍，建设１００所标准化学校，力争高中阶段教育毛入学率达７０％。鼓励和规范民办教育。统筹抓好各类人才的引进、培养和使用，筹建市高级人才库，让优秀人才脱颖而出。

  统筹抓好医疗卫生和人口计生工作。深入推进新型农村合作医疗，加强农村卫生人才队伍建设，落实公共卫生补助机制。开工建设马山医院新病区、医疗废弃物处置中心，完成市第一人民医院改扩建、２０所乡镇卫生院新建和改扩建、６８所村卫生室、３０个社区卫生机构建设任务。大力开展城乡爱国卫生运动，扩大农村免费公共卫生服务范围，突出抓好重大疾病防治和应急处置工作。加强人口和计划生育工作，搞好普惠性政策和计划生育家庭优惠政策的衔接，加快计划生育服务阵地建设。

  全方位促进就业再就业。支持自主创业、自谋职业和灵活就业，建成农民回乡创业园２个。完善就业公共服务体系，积极做好外出务工返乡人员、高校毕业生、退伍军人、失地农民、残疾人就业，动态消除“零就业家庭”，新增农���劳动力转移就业３万个，实现下岗人员再就业1.5万人。加强创业、就业和再就业培训，全年完成各类职业培训２万人。建立新型劳动关系，确保劳动违法案件、劳动争议案件限时办结。

  进一步提高社会保障水平。积极推行新型农村合作医疗市级统筹，争取参合率达９０％以上；完善城镇职工基本医疗保险，切实解决异地就医难问题；继续降低城镇居民基本医疗保险缴费标准，提高报销水平，扩大门诊范围，提高医疗保险“三张网”的覆盖率。调整完善企业退休人员、被征地农民养老保险政策，探索农村社会养老保险，适时提高协保人员待遇标准。进一步完善“五保合一”，全年完成参保人数134.5万人，征缴率均达９０％以上。加大城乡社会救助力度，实现城乡最低生活保障应保尽保，农村五保集中供养率达３０％，改扩建农村敬老院１０所。大力发展社会福利和慈善事业，抓好市社会福利院综合楼、市流浪未成年人救助保护中心建设，筹建市级老年公寓。完善成品粮油等生活必需品预警、储备、调控办法。

  巩固提升民生工程。进一步加大投入、巩固成果、健全机制，让人民群众得到更多实惠。从今年开始，全市民生工程扩大到３５项，其中省民生工程２８项、市民生工程７项，投入财政资金６亿多元，惠及２２０万人。积极整合各类事关民生的政策资源，建立多元筹资机制和考核评价管理制度。推进民生工程与社会保障体系建设、社会事业发展的有效衔接，建立健全改善民生的长效机制。

  （三）强化社会管理，维护城乡社会稳定

  扎实推进平安淮南建设。健全社会治安防控体系、严打整治体系，完善矛盾纠纷预防化解机制和治安形势评估机制，深入开展平安创建活动。依法防范和打击违法犯罪活动，保障人民生命财产安全，确保社会大局稳定。做好信访工作，完善信访制度。进一步畅通群众诉求渠道，依法依规解决群众反映的突出问题，努力实现信访减量退位。继续整顿规范市场秩序，重拳打击非法传销、非法集资、商业欺诈和制假售假等违法行为。加强公共突发事件应急管理体制机制法制建设，提高保障公共安全和处置突发事件的能力。

  切实抓好安全生产。加大源头治理力度，遏制重特大事故发生。坚持不懈地抓好煤矿安全和淮河水灾两大核心隐患的综合治理，抓好各类安全事故隐患的排查和整治，完善预报、预警、预防和应急救援体系。依法加强监管，从严从快查处安全生产事故，对事故责任人进行严肃追究。强化农产品安全建设和食品药品监管，切实让人民群众吃得放心、用得安心。

  全面加强民主法制建设。各级政府自觉接受人大及其常委会的法律监督，自觉接受政协的民主监督，接受新闻舆论和公众的社会监督，坚持重大事项向人大报告、与政协协商。推进依法治市，开展法治城市、法治县区创建活动。发挥享有地方立法权的优势，积极开展住房保障、城市供热等立法工作。坚持政府信息公开和政府新闻发布会制度，继续办好政风行风热线。着力改善社区办公条件，积极开展和谐社区创建活动。通过财政转移支付等途径，加强农村基层组织建设。加快山南新区村镇调整，加强规划地名规范化管理。搞好国防教育、人民防空、民兵预备役工作，加强市民兵训练基地建设，提高国防动员能力。广泛开展双拥共建活动，争创省级双拥模范城“七连冠”。大力推进老龄、妇女儿童、残疾人工作，做好广播电视、民族宗教、防震减灾、地方志、统计、气象和档案等工作。

**六、加大以开放促改革、以改革促发展力度**

  面对金融危机，我们必须坚定不移地实施大开放，在更高层次、更大范围、更广领域利用资源、抢占先机，必须坚定不移地推进各项改革，启动内力、激发活力。

  深入推进合淮同城化。积极构建合淮同城化合作机制，完善落实两市合作框架协议和专项合作协议，加速融入省会经济圈。一是加快规划对接，完成并组织实施合淮同城化总体规划、合淮工业走廊规划、大交通及能源规划、区域城乡一体化规划。二是加快交通对接。积极争取合淮蚌铁路客运专线开工，做好九龙大道（九龙岗至水家湖接合肥双凤大道）、舜耕大道（十五中转盘至房山集接合肥蒙城北路）、淮南大道（安成铺转盘至曹庵接206国道）等3条公路改扩建开工准备，推动引江济淮工程前期工作。启动城市快速公交系统规划建设，构建合淮城际公交体系。三是加快产业对接。积极培育产业集聚区、高载能工业园、大型物流园区。与合肥联手，积极争取省里把合淮工业走廊作为承接长三角产业转移示范区。四是加快市场对接。推动劳动力市场、人才市场、产权交易市场互联，实现旅游资源、教育资源、医疗资源互通，市场准入资质、标准、认证、检测互认。

  着力加快重点领域和关键环节改革。继续推进淮南矿业集团、淮化集团Ａ股上市，指导培育３户企业中小板上市。继续推进皖淮机械厂实施军民品分离，加快淮化集团主辅分离、辅业改制，争取皖纸公司实行资产重组，加快市属出中心企业资产处置，积极争取进入省第一批厂办大集体企业改革试点。妥善处理国有企业留守处问题。制定加强国有资产监管的意见和产权交易管理办法。积极做好不良金融资产打包处置工作。完成工商系统脱钩改革任务。推进供销合作社改革发展。继续推进农村综合改革和集体林权配套改革。深化土地使用制度改革，落实经营性土地和工业用地招拍挂制度，加快建立农村土地承包经营权流转市场，发展多种形式的适度规模经营。切实加强土地执法，严格耕地保护措施。全面推进建设用地置换，新增建设用地4000亩。加大土地整理复垦力度，实现耕地占补平衡，开发整理土地9000亩，逐步调整完善市县区财政体制和财政支出结构，加大对农村、民生、公共服务等领域投入。继续深化公用事业、公益事业价格改革。进一步完善统计管理体制改革。

  强力提升对内对外合作水平。转变外贸增长方式，优化出口产品结构，力争完成外贸进出口总额1.2亿美元。扩大同省内城市的交流，巩固友城关系。实施“走出去”战略和外经贸“孵化器”工程，支持有条件的企业开展对外工程承包和劳务合作，支持中央和省属单位、大专院校、科研院所、驻淮部队在淮发展。完善外经、外贸、外事、外智、外宣相统筹的大开放格局。

**七、加强政府管理创新**

  面对新形势新要求，政府重任在肩。我们务必把思想和行动统一到中央、省委和市委的决策部署上来，务必保持奋发有为的精神状态，以强烈的事业心和责任感，扎实做好党和人民交给我们的各项工作。

  继续解放思想。牢固树立科学的发展观、正确的政绩观和正确的群众观，全面提升学习力、实践力和执行力，真正在思想上具备科学发展的境界，在工作上树立科学发展的标准，在作风上符合科学发展的要求，在成效上体现科学发展的水平。勇于用新的理念、观念指导实践，善于用新的思维、思路破解难题，敢于用改革、开放、创新的举措谋求突破，强化“七破七立”，建立“八个机制”，努力在日趋激烈的区域竞争中，在日益复杂的环境中，率先发展、科学发展、和谐发展。

  推进简政放权。深化行政管理体制改革，把政府经济管理工作的重心转移到提供优质服务和创造良好环境上来。适时启动政府机构改革，做好经营性事业单位转企改制，进一步优化政府机构设置，努力解决机构重叠、职能交叉、权责脱节、政出多门的问题。进一步加大向县区放权力度，继续推进市、县区、乡镇三级联动服务体系建设。加强“信用淮南”建设，完善信用体系。加强“数字淮南”建设，启动市直机关网上协同办公系统、电子监察系统建设，拓宽网上审批范围，提高在线办事水平。努力把淮南打造成为审批环节最少、服务意识最强、办事效率最高的地区之一。

  强化闭环管理。坚持决策、执行、监督、反馈等环节的有机统一，逐步在政府、部门和行业内部，在政府与部门之间、部门与部门之间形成闭环，确保各项工作件件有落实、事事有回音、人人有职责。坚持科学决策和民主决策，进一步完善重大问题调查研究、集体决策、专家咨询、社会公示和听证制度，积极推行“一线工作法”，做到决策在一线落实、问题在一线解决、创新在一线体现、成效在一线检验。坚持一级抓一级、一环扣一环，凡是政府作出的承诺、决定的事项，必须不折不扣地落到实处，确保政令畅通。

  坚持廉洁从政。认真贯彻党风廉政建设责任制，着力完善惩治和预防腐败体系。巩固反腐倡廉制度建设推进年活动成果，严格落实各项规章制度，凭制度用权、靠制度办事、以制度管人，让权力在监督下实施、在阳光下运行。坚持勤俭节约，坚决反对铺张浪费和奢靡之风。加大对工程承包、土地出让、政府采购、资源开发、产权交易和医药购销等重点领域的治理力度，坚决纠正部门和行业不正之风。扎实开展优化经济发展环境集中整治活动，为企业依法经营创造宽松环境。进一步强化审计、监察工作，严厉查处各类违法违纪案件。加快建设风清气正的政治生态环境，着力营造团结和谐的干事创业氛围。

  各位代表，做好今年各项工作，实现预期发展目标，既有挑战，又有机遇。我们坚信，任何困难都改变不了我们创造美好生活的强烈愿望，任何挑战都阻挡不了我们加快奋力崛起的坚定步伐！让我们紧密团结在以胡锦涛同志为总书记的党中央周围，高举中国特色社会主义伟大旗帜，在市委的坚强领导下，励精图治，奋发进取，以推进科学发展、加速淮南崛起的优异成绩迎接新中国成立六十周年！
